# HPK1 Associates with SKAP-HOM to Negatively Regulate Rap1-Mediated B-Lymphocyte Adhesion

**DOI:** 10.1371/journal.pone.0012468

**Published:** 2010-09-01

**Authors:** Sebastian Königsberger, Doris Peckl-Schmid, Nadja Zaborsky, Irene Patzak, Friedemann Kiefer, Gernot Achatz

**Affiliations:** 1 Department of Molecular Biology, University of Salzburg, Salzburg, Austria,; 2 Lincoln's Inn Fields Laboratories, Cancer Research UK London Research Institute, London, United Kingdom,; 3 Department Vascular Cell Biology, Max Planck Institute for Molecular Biomedicine, Münster, Germany; University of Miami, United States of America

## Abstract

**Background:**

Hematopoietic progenitor kinase 1 (HPK1) is a Ste20-related serine/threonine kinase activated by a range of environmental stimuli including genotoxic stress, growth factors, inflammatory cytokines and antigen receptor triggering. Being inducibly recruited to membrane-proximal signalling scaffolds to regulate NFAT, AP-1 and NFκB-mediated gene transcription in T-cells, the function of HPK1 in B-cells to date remains rather ill-defined.

**Methodology/Principal Findings:**

By using two loss of function models, we show that HPK1 displays a novel function in regulating B-cell integrin activity. Wehi 231 lymphoma cells lacking HPK1 after shRNA mediated knockdown exhibit increased basic activation levels of Ras-related protein 1 (Rap1), accompanied by a severe lymphocyte function-associated antigen-1 (LFA-1) dependent homotypic aggregation and increased adhesion to intercellular adhesion molecule 1 (ICAM-1). The observed phenotype of enhanced integrin activity is caused downstream of Src, by a signalling module independent of PI3K and PLC, involving HPK1, SKAP55 homologue (SKAP-HOM) and Rap1-GTP-interacting adaptor molecule (RIAM). This alters actin dynamics and renders focal adhesion kinase (FAK) constitutively phosphorylated. Bone marrow and splenic B-cell development of HPK1^−/−^ mice are largely unaffected, except age-related tendencies for increased splenic cellularity and BCR downregulation. In addition, naïve splenic knockout B-cells appear hyperresponsive to a range of stimuli applied *ex vivo* as recently demonstrated by others for T-cells.

**Conclusions/Significance:**

We therefore conclude that HPK1 exhibits a dual function in B-cells by negatively regulating integrin activity and controlling cellular activation, which makes it an interesting candidate to study in pathological settings like autoimmunity and cancer.

## Introduction

Integrin regulation is essential in mediating homeostatic lymphocyte features like adhesion, migration and immune synapse formation [1]–[3]. While being kept in an inactive state in resting cells, inside-out activation signals from the BCR, TCR or chemokine receptors facilitate the binding of the β integrin chain to talin [4]. As a consequence, membrane distribution and/or affinity for ligands inducibly change and concomitant outside-in integrin signalling can lower antigenic activation thresholds for B- and T-cells [5], [6]. Recent studies propose B-cells to mainly recognize antigen in the form of immune complexes bound to Fc or complement receptors on e.g. follicular dendritic cells [7]. If the signals provided in the context of this interaction exceed a certain activation threshold, arrest of the B-cell eventually allows the formation of a peripheral supramolecular activation cluster composed of lymphocyte function-associated antigen-1 (LFA-1) and very late antigen-4 (VLA-4) integrins, which subsequently serve as a docking platform to increase the duration of the synapse and ensure proper B-cell activation [6], [8].

The small GTPase Ras-related protein 1 (Rap1) belongs to the Ras superfamily and has a pivotal function in lymphocyte integrin activation [9]–[11]. By binding downstream effectors like regulator for cell adhesion and polarization enriched in lymphoid tissues (RAPL) and Rap1-GTP-interacting adaptor molecule (RIAM), Rap1 connects to talin [12] and induces lymphocyte polarization by distributing LFA-1 to the leading edge, which is indispensable for cellular adhesion and migration [13], [14]. In T-cells, the two constitutively associated adaptor molecules adhesion- and degranulation-promoting adaptor protein (ADAP) and Src-kinase-associated phosphoprotein of 55 kDa (SKAP55) were shown to be of critical importance in translating signals from the TCR via Rap1 activation to LFA-1 and VLA-4 integrins [15]–[19]. Although ADAP and SKAP55 are not expressed in B-cells, a SKAP55 homologue (SKAP-HOM) broadly expressed in the hematopoietic system fulfils similar tasks in B-cells and macrophages [20]–[22].

In the current paper we demonstrate that HPK1, a Ste20-related serine/threonine kinase triggering the SAPK/JNK pathway [23], [24], constitutively associates with SKAP-HOM in Wehi 231 cells, forming a ternary complex of HPK1, SKAP-HOM and RIAM. Wehi 231 cells lacking HPK1 expression after shRNA mediated knockdown display substantially increased LFA-1 mediated homotypic aggregation and adhesion to ICAM-1. This behaviour is caused by an upregulation of Rap1-GTP in unstimulated cells, leading to dysregulated actin dynamics and enhanced focal adhesion kinase (FAK) activity. We further show that HPK1^−/−^ mice develop normal bone marrow and splenic B-cell subset counts but exhibit an increase in B-cell reactivity, suggesting that HPK1 limits the actions emanating from the SKAP-HOM adaptor complex needed for proper B-cell activation and adhesion.

## Results

### HPK1 negatively regulates B-cell adhesion

**Figure 1:**
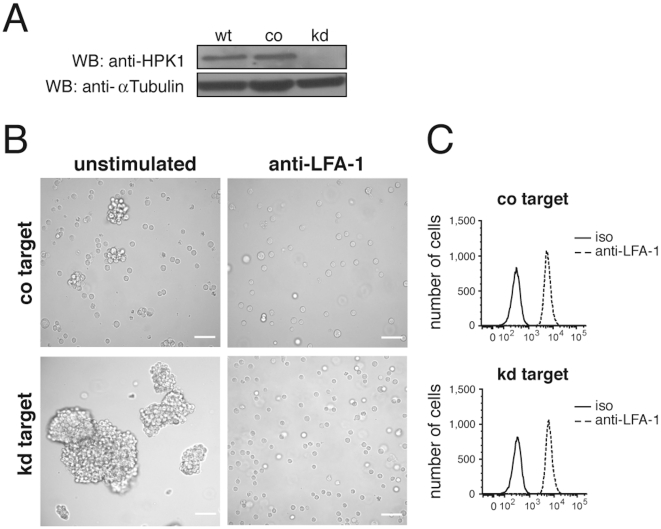
Homotypic aggregation of HPK1 knockdown Wehi 231 cells. (A) HPK1 protein expression in Wehi 231 wild type (wt), control (co) and knockdown (kd) cells determined by anti-HPK1 Western blotting. (B) Homotypic aggregation of Wehi 231 kd transfectants compared to transfection controls; cluster formation was blocked by adding anti-LFA-1 antibody [30 µg/ml] for 17 h into the culture medium (Zeiss Axiovert 135, scale bar: 50 µm). (C) Flow cytometric analysis (FACS Canto II) of Wehi 231 co and kd cell LFA-1 surface expression; iso, isotype control.

**Figure 2:**
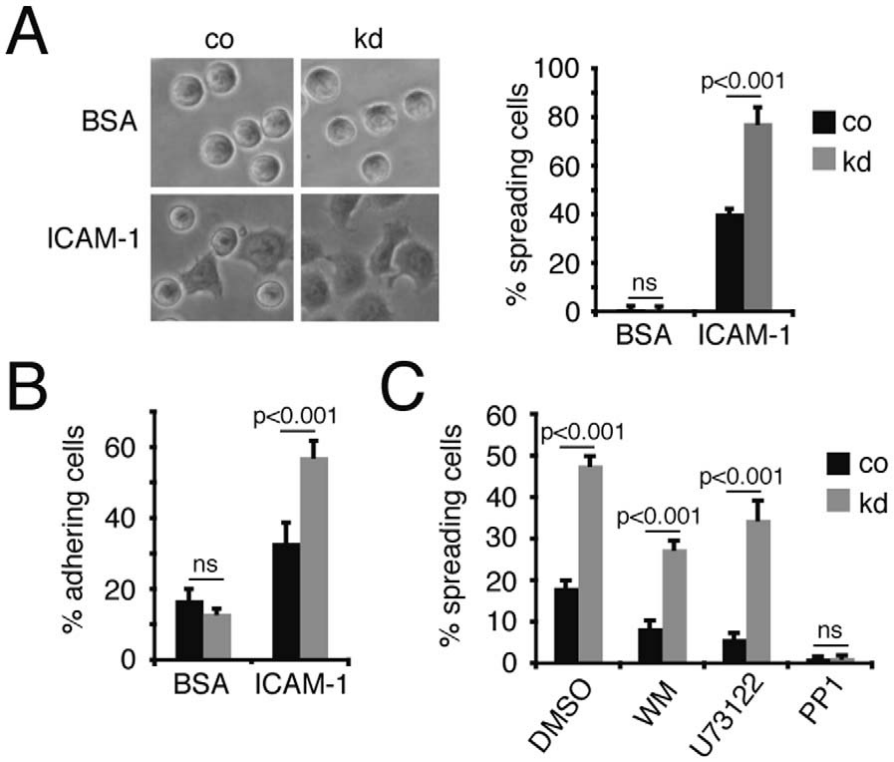
HPK1 negatively regulates adhesion to ICAM-1 independent of PI3K and PLC. (A) Left: Phase contrast images showing Wehi 231 co and kd cell spreading on BSA and ICAM-1 (Nikon Eclipse, 20×) after 30 min of adhesion; right: percentage of spread, phase dark co and kd cells on ICAM-1. (B) Percentage of CFSE labelled co and kd cells adhering to BSA or ICAM-1 after manually applied shear stress. (C) Percentage of co and kd cells adhering to ICAM-1 after pharmacological inhibitor treatment (100 nM Wortmannin, 1 µM U73122, 100 µM PP1); results are representative for three (A,C) or six (B) independent experiments and are presented as means ± SD (Student's t); ns, not significant.

For the analysis of HPK1 deficiency in B-cells the immature B-cell line Wehi 231 was chosen as target for RNA interference. shRNA mediated silencing reduced HPK1 protein expression by approximately 95% (Figure 1A). While control (co) target cells showed a wild type like growth behaviour of single cells and small homotypic aggregates (Figure 1B, upper left), Wehi 231 HPK1 knockdowns (kd) formed large cellular clusters (Figure 1B, lower left) phenocopying the general growth of proliferating primary B-cells *in vitro*. As these cellular interactions are critically dependent on integrins, we speculated about an impact of HPK1 on integrin regulation. Blocking α_L_β_2_ integrin with an anti-LFA-1 specific antibody applied to the medium for 17 h completely interfered with co and kd cell aggregation (Figure 1B, right), suggesting that increased cellular clustering was caused by augmented LFA-1 activity, whereas the lack of HPK1 had no influence on total membrane LFA-1 expression as measured by flow cytometry (Figure 1C). To further monitor the adhesion behaviour of transfectants under conditions of manually applied shear stress, co and kd cells were subjected to static adhesion on ICAM-1/Fc coated plates. Wehi 231 cells lacking HPK1 exhibited a twofold increase (p<0.001) in adhesion to ICAM-1/Fc compared to co cells (Figure 2B) and showed a significantly higher percentage (p<0.001) of spreading cells (Figure 2A).

Inside-out activation of integrins triggered by the BCR was shown to be regulated by the sequential activation of signalling mediators including Src family kinases, PI3K, PLCγ2, Btk, guanosine nucleotide exchange factors like e.g. Vav1/2 as well as small GTPases of the Rho and Ras family [9], [25], [26]. To determine if the effect of increased integrin activity observed in the Wehi 231 kd cells is caused by alterations in the activity of one of the factors mentioned, we inhibited Src kinases (PP1), PI3K (Wortmannin) and PLC (U73122) as central membrane proximal players by the use of pharmacological treatment. Figure 2C displays the highest inhibitor concentrations applied which still maintained cellular viability and were assumed to have the strongest effect on target activity. Wortmannin (100 µM) and U73122 (1 µM) treated co and kd cells showed a highly significant difference (WM co: 8.54%±1.72%; kd: 27.52%±1.95%; U73122 co: 5.91%±1.31%; kd: 34.66%±4.46%) in adhesion as observed for DMSO controls (co: 18.18%±1.76%; kd: 47.72%±2.15%), suggesting that although PLCγ2 and PI3K contribute to overall adhesion, they are not causative for the observed increase in LFA-1 activity. Interfering with Src kinase activity (100 nM PP1) completely abolished adhesion in both co (1.21%±0.39%) and kd (1.31%±0.57%) cells, which likely positions the regulatory function of HPK1 downstream of Src.

### HPK1 associates with the SKAP-HOM – RIAM complex and limits Rap1 activation

In T-cells, the ADAP-SKAP55 adaptor complex was shown to be of critical importance in T-cell adhesion by transducing signals from the TCR to integrins [15]–[19]. SKAP55 is known to interact with RIAM [18], an adaptor molecule previously shown to provide a physical link between the small GTPase Rap1 and talin [12] which binds to the β integrin chain as a central step in changing integrin affinity [4].

**Figure 3:**
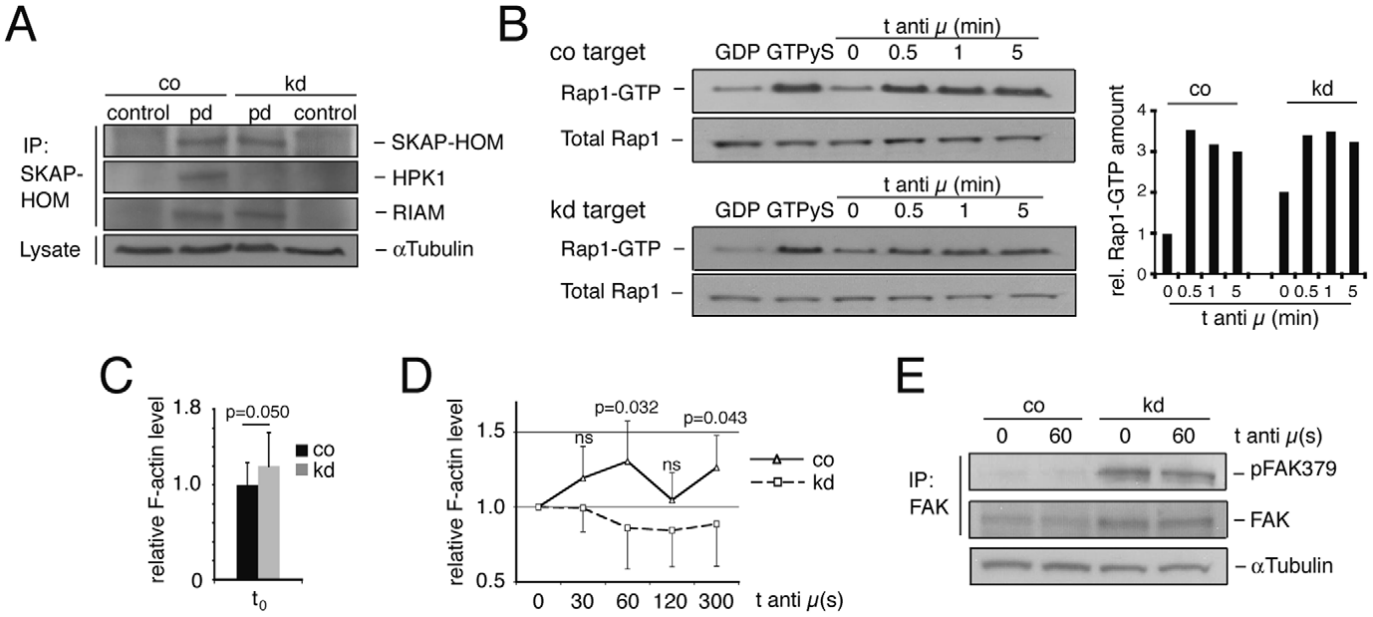
HPK1 associates with SKAP-HOM and negatively regulates Rap1 activation. (A) SKAP-HOM was immunoprecipitated in unstimulated Wehi 231 co/kd cells and pulldowns were detected for SKAP-HOM, HPK1 and RIAM expression by Western blotting; plots are representative for four independent experiments. (B) Left: Active Rap1-GTP of untreated and anti-IgM F(ab')2 (anti µ) stimulated co and kd cells was pulled down with Ral GDS RBD agarose and analysed together with total Rap1 levels by anti-Rap1 Western blotting; quantification of band intensities was performed by normalizing total Rap1 levels of lysates by densitometric evaluation (ImageJ) and all time points of Rap1-GTP samples were compared to the control target at t_0_ = 1; GDP/GTP: negative/positive control; right: graph representative for three independently performed experiments. (C) F-actin levels (means ± SD; Student's t; n = 6) of untreated co and kd cells measured by Phalloidin-FITC flow cytometric staining. (D) F-actin levels (means ± SD; Student's t; n = 6) of anti-IgM F(ab')2 (anti µ) stimulated co and kd cells. (E) Untreated and anti-IgM stimulated FAK immunoprecipitates of Wehi 231 co and kd cells were detected for total FAK and pFAK379 expression by Western blotting (n = 3); ns, not significant; pd, pulldown.

As it remains unclear whether an ADAP homologue in B-cells exists, we asked if HPK1 links to the SKAP55 homologue SKAP-HOM expressed in B-cells. Indeed, HPK1 co-immunoprecipitated with SKAP-HOM in Wehi 231 cells (Figure 3A, upper two panels), providing a novel physical link to a B-cell adhesion regulatory module. In addition, as true for SKAP55 in T-cells [18], SKAP-HOM interacted with RIAM (Figure 3A, third panel). Because HPK1 and RIAM both physically link to SKAP-HOM and the observed growth of the Wehi 231 kd cells resembled the reported behaviour of Rap1V12 transfected cell lines [27], we investigated Rap1-GTP expression in unstimulated and anti-IgM (anti µ) stimulated transfectants. In unstimulated knockdown cells, the initial amount of Rap1-GTP was twice as high as in the control cells, whereas antigen receptor stimulation led to comparable Rap1-GTP amounts in knockdown and control clones (Figure 3B), suggesting a negative regulatory role of HPK1 on B-cell adhesion by limiting Rap1 activation.

### Lack of HPK1 affects actin polymerization and focal adhesion kinase activity

RIAM was shown to interact with Profilin and Ena/VASP proteins to regulate actin polymerization and cell spreading [28]. We hypothesized that the increased amount of active Rap1 found in the kd cells could affect the activity of the downstream adaptor RIAM and therefore change cellular F-actin levels. Wehi knockdowns stained for F-actin by the use of Phalloidin-FITC showed a higher basal F-actin level (Figure 3C) than co cells, a phenotype reported for cells with increased RIAM activity [28], which possibly explains the enhanced spreading of kd cells. In addition, kd cells exhibited significantly less *de novo* actin polymerization upon anti-IgM F(ab')2 stimulation (Figure 3D) which could be the result of uncoupling the actin regulatory SKAP-HOM – RIAM complex from coordinated signals through the BCR.

An additional downstream target of Rap1 activity is the focal adhesion kinase (FAK). FAK expression was shown for Wehi 231 and A20 cells as well as for activated B-cells, where the protein was suggested to colocalize with LFA-1 and VLA-4 in a manner critically dependent on actin dynamics to regulate cell spreading [29]. In addition, FAK was shown to associate with the Arp2/3 complex, therefore linking integrin signalling directly with actin polymerization [30]. The loss of HPK1 in Wehi 231 kd cells led to a constitutive and profound increase in the amount of active FAK phosphorylated on Y379 (pFAK379), while anti-IgM (anti µ) stimulation had no influence on pFAK379 levels either in co or kd cells (Figure 3E), which indicates an integrin-mediated FAK activation mechanism independent of the BCR. Thus, we provide evidence that through the lack of HPK1, enhanced Rap1 activity affects actin dynamics and the phosphorylation of the focal contact component FAK.

### Murine B-cell development is largely unaffected by the lack of HPK1

**Figure 4:**
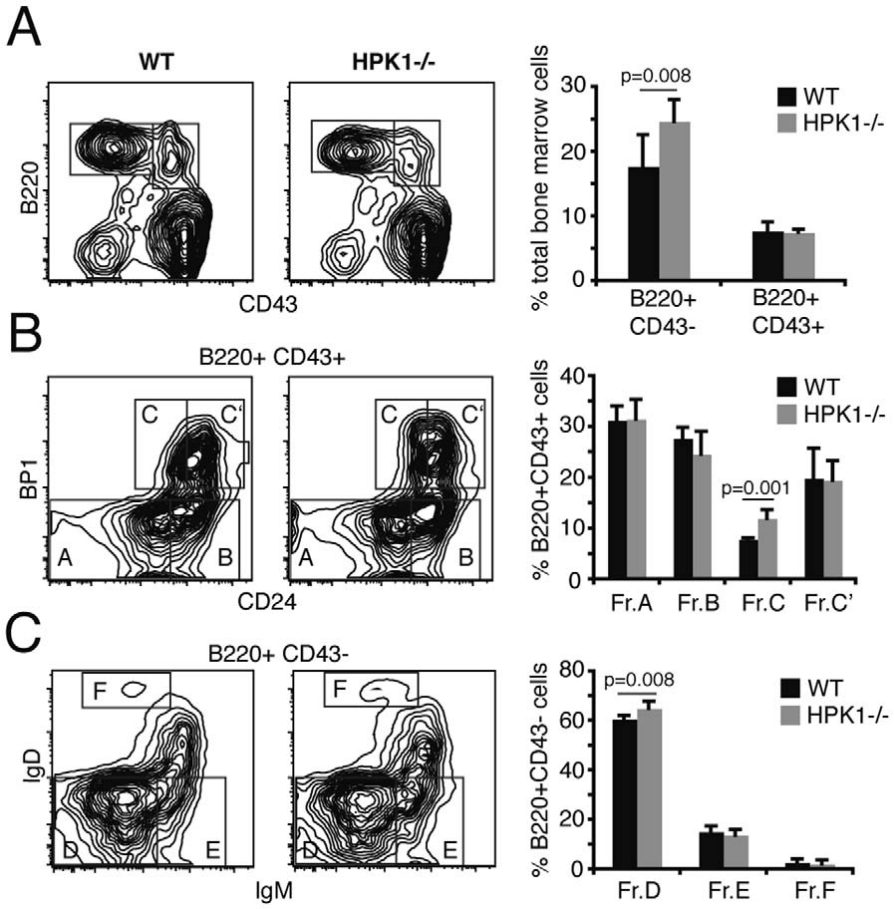
Bone marrow B-cell development of HPK1^−/−^ mice. (A) Total bone marrow of 7 weeks old WT and HPK1^−/−^ mice stained with CD43/B220 to delineate early and late B-cell fractions; cells were further stained with (B) BP1/CD24 for pro-pre B-cells stages or (C) IgM/IgD for immature/transitional fractions; right: graphs are representative for 5 independent experiments and results are presented as means ± SD (Student's t).

**Figure 5:**
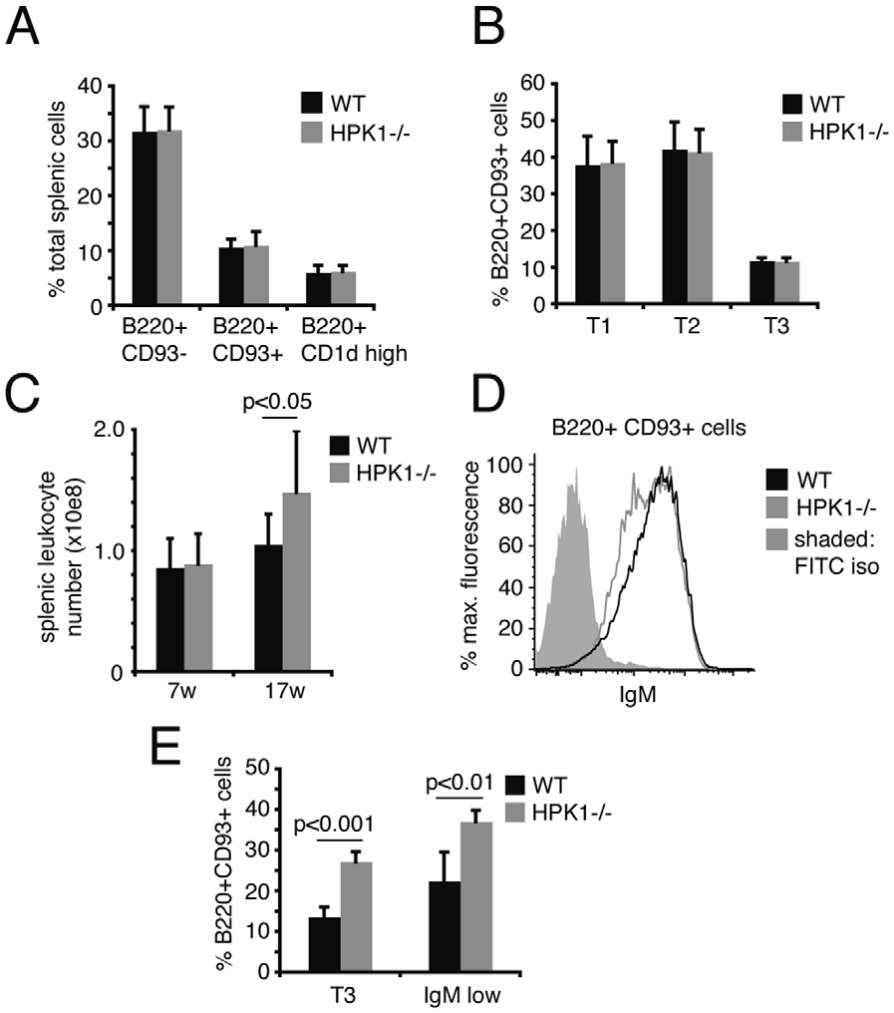
HPK1 deficiency has a late onset effect on splenic leukocyte numbers and surface IgM levels. (A,B) Splenic cells of 7 weeks old wt and HPK1^−/−^ mice were stained with distinct cell surface markers to delineate peripheral B-cell development (A: mature B220^+^ CD93^−^, transitional (T) B220^+^CD93^+^, marginal zone B220^+^CD1d^+^; B: T1 B220^+^ CD93^+^ IgM^+^ CD23^−/low^, T2 B220^+^ CD93^+^ IgM^+^ CD23^+^, T3 B220^+^ CD93^+^ IgM^low^ CD23^+^; graphs: means ± SD; Student's t; n = 5). (C) Total splenic leukocyte counts of 7 and 17 weeks (w) old WT and HPK1^−/−^ mice. (D) Surface IgM levels of aged (40 w) WT and HPK1^−/−^ mice pre-gated on B220^+^CD93^+^ transitional B-cells (graph representative for 4 independent experiments). (E) Percentage of aged (40 w) wt and HPK1^−/−^ T3/IgM^low^ cells gated on total transitional B220^+^CD93^+^ cells (graphs: means ± SD; Student's t; n = 4); iso, isotype control.

Recently, HPK1^−/−^ mice were reported to have normal numbers of B220^+^ cells and B-cell/T-cell ratios in peripheral lymphoid organs [31]. In line with these findings, our detailed analyses of B-cell subsets in the bone marrow showed that mice lacking HPK1 are only mildly affected and develop largely normal cell numbers. We observed a slight enlargement of the Hardy fraction C, composed of B220^+^CD43^+^BP1^+^HSA^int^. pro-B cells (Figure 4B) which are known to be in close contact with the stromal environment via CD44 and VLA-4 to ensure functional pro-pre-B-cell transition [32]. This leads to an increase in the small pre- (Figure 4C, Fr.D) and the mature B220^+^CD43^−^ B-cell population (Figure 4A). Nevertheless, the slightly increased cellularity of the mature B-cell fraction in the bone marrow of HPK1^−/−^ mice did not significantly affect the numbers of transitional (T1: B220^+^ CD93^+^ IgM^+^ CD23^−/low^, T2: B220^+^ CD93^+^ IgM^+^ CD23^+^, T3: B220^+^ CD93^+^ IgM^low^ CD23^+^) and mature (B220^+^ CD93^−^)/marginal zone (B220^+^ CD1d^+^) B-cells in the spleen (Figure 5A,B). Interestingly, older HPK1^−/−^ mice (17 w) exhibited increases in splenic leukocyte numbers compared to wt mice (Figure 5C). Aged (40 w) HPK1^−/−^ mice showed reductions of surface IgM density in transitional B-cells (Figure 5D) which leads to a significant increase in the transitional 3/IgM^low^ fraction (Figure 5E), a phenotype that in an attenuated form reflects the B-cell development observable in anti-HELtg/HELtg mice [33]. The findings of Shui et al. [31] additionally stated a stronger overall increase in specific immunoglobulin levels after T-cell dependent protein antigen administration in HPK1^−/−^ mice, which was suggested to be the result of cognate T-cell hyperreactivity. An alternative explanation of the described B-cell hyperreactivities could be a cell intrinsic defect in limiting Rap1-dependent integrin activation known to be of critical importance in refining antigenic activation thresholds.

### Altered B-cell reactivity and BCR dynamics in HPK1^−/−^ mice

**Figure 6:**
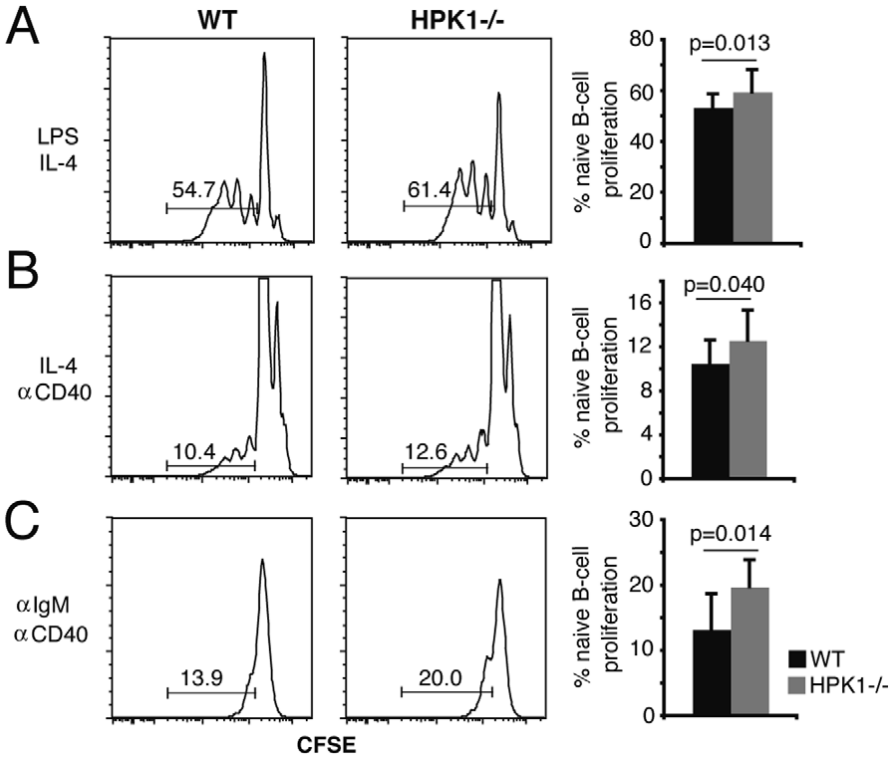
HPK1 negatively regulates B-cell proliferation. Naïve splenic B-cells of 7 weeks old WT and HPK1^−/−^ mice were labelled with CFSE and stimulated with (A) LPS (25 µg/ml) and IL-4 (5 ng/ml), (B) IL-4 and anti-CD40 (10 µg/ml) or (C) anti-IgM F(ab')2 (5 µg/ml) and anti-CD40 for 72 h; percentages of proliferating B-cells were determined by gating on live (DAPI^−^) cell fractions and determining signal cut off by the use of unstimulated samples; results are representative for 6 independently performed experiments (Student's t).

To assess B-cell reactivity in HPK1^−/−^ mice naïve splenic B-cells were purified untouched, CFSE labelled and subjected to *in vitro* proliferation by the use of different B-cell stimuli. Anti-IgM (5 µg/ml)/anti-CD40 (10 µg/ml) as well as LPS (25 µg/ml) and LPS (25 µg/ml)/IL-4 (5 ng/ml) induced proliferation was significantly increased in HPK1^−/−^ mice (Figure 6A,C and Figure S3), while anti-CD40/IL-4 stimulation resulted in the weakest differences, suggesting a negative regulatory effect of HPK1 on BCR- and TLR-4 mediated B-cell activation operating independently on CD40 signalling (Figure 6B and Figure S3). We further speculated whether the phenotype of altered actin dynamics, as shown for the Wehi knockdowns, as well as the reported association of HPK1 with the actin regulatory protein HS1 [34]–[37], might cause altered actin-mediated BCR uptake kinetics. Therefore we performed anti-IgM-FITC internalization assays with primary splenic cells. Splenic IgM^+^ cells of HPK1^−/−^ mice displayed an unsignificant tendency (n = 4) for a slower BCR endocytosis rate (Figure S1A), an effect that became more obvious by knocking down HPK1 expression in CH27 cells (Figure S1C). CH27 B- lymphoma cells display a transitional maturation phenotype (IgM^+^ IgD^+^) and, in contrast to Wehi 231 cells, efficiently internalize antigen receptors upon stimulation. HPK1 kd CH27 cells displayed significantly slower IgM uptake kinetics after 5 and 10 min on 37°C compared to the co target (Figure S1B), an effect gradually lost with increasing HPK1 concentration as CH27 clones with a less efficient knockdown showed internalization rates insignificantly different from co cells (data not shown). Thus, we show that while B-cell developmental processes are largely unaffected by the lack of HPK1, antigen receptor- and TLR-4-induced proliferation is limited by the action of HPK1.

## Discussion

**Figure 7:**
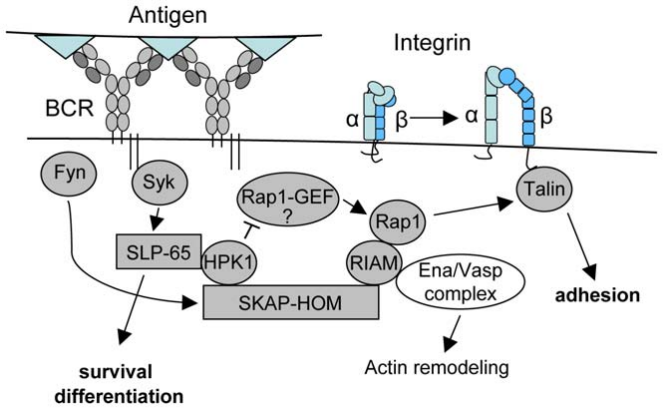
Model for HPK1 mediated negative regulation of B-cell integrin activity. Antigen receptor stimulation triggers the association of HPK1 with the two central B-cell adaptors SLP-65 [46] and likely SKAP-HOM, which regulate cellular survival/differentiation and adhesion. In this context, we suggest HPK1 to act as a dual regulator by shaping SLP-65 mediated signals and restricting Rap1 activation required for B-lymphocyte adhesion; SLP-65, Src homology 2 domain-containing leukocyte protein of 65 kDa; Syk, spleen tyrosine kinase.

In this paper we provide evidence for a novel function of HPK1 in regulating B-lymphocyte adhesion. By associating with the SKAP-HOM – RIAM module, HPK1 limits Rap1-GTP-mediated signals aiming to upregulate integrin activity upon antigen receptor triggering (Figure 7). B-cells lacking SKAP-HOM were reported to be deficient in β1 and β2 integrin-mediated adhesion and exhibit a mild decrease in anti-IgM induced *in vitro* proliferation, while displaying normal membrane-proximal signalling upon BCR triggering and systemic immunoglobulin responses to T-dependent antigens [22]. HPK1^−/−^ B-cells intriguingly oppose this phenotype, as in our hands they showed wild type-like specific serum Ig levels after administration of T-dependent antigen (Figure S2) but enhanced *in vitro* proliferation upon anti-IgM/IL-4 and LPS stimulation. Interestingly, a similar immunization approach on the C57/BL6 background done by Shui et al. [31] showed significant differences in all Ig isotype levels investigated. Our hypothesis is that the negative regulatory function of HPK1 might vary with the genetic background and in our study is linked to a rather age-related effect. In this regard one might speculate whether a biased negative selection in the bone marrow due to a change in signal quality from the BCR could possibly lead to a larger pool of autoreactive peripheral T3/IgM low B-cells as recently described in mice [33]. The caveat of the knockout model addressed is that the actions of HPK1 could be compensated on the cellular level by e.g. additionally affecting regulatory T-cell function. The involvement of HPK1 in various signalling pathways implies a complex pattern of regulation which might partially account for the controversial results gained from T-cell studies on HPK1-mediated regulation of AP-1 activity and IL-2 transcription [23], [38]–[41]. This necessitates the generation of cell specific and inducible knockout approaches to further delineate the exact nature of regulation in a systemic setting.

Although we found an upregulation of Rap1-GTP levels in cells lacking HPK1, the question of the guanosine nucleotide exchange factor responsible for the enhanced Rap1 activation in this setting is still unclear. A possible way to dampen Rap1 activity could be the reported association of HPK1 with CrkL [39], which was shown to bind the Rap1 exchange factor C3G [42] and localizes to the immunological synapse in a WAVE2 dependent manner [43]. Our results further indicate a function of HPK1 in the indirect control of cellular F-actin dynamics possibly by limiting the action of the downstream Rap1 effector RIAM.

Taken together, further studies will be needed to in deep characterize how HPK1 mediates its negative regulatory function on both Rap1-mediated B-cell integrin activation as well as cellular proliferation.

## Materials and Methods

### Antibodies and reagents

Anti-IgM F(ab')2 (Zymed), anti-B220-APC (RA3-6B2; BD), anti-IgM-FITC/PE (R6-60.2; BD), anti-CD43-FITC (S7; BD), anti-BP1-PE (6C3; BD), anti-HSA-PE-Cy7 (M1/69; Biolegend), anti-IgD-Biotin (11–26, Southern Biotech), Streptavidin-Cy7 (eBio), anti-CD23-PE (B3B4; BD), anti-CD93-PE-Cy7 (AA4.1; eBio), anti-CD1d-FITC (1B1; BD), anti-CD16/CD32 (2.4G2; BD), anti-HPK1 (N19; Santa Cruz), anti-RIAM (Proteintech Group), anti-SKAP-HOM (C17; Santa Cruz), anti-SKAP-HOM (Millipore), anti-alpha Tubulin (DM1A; Abcam), anti-FAK (14; BD), anti-FAK (C903; Santa Cruz), anti-pFAK379 (77; BD), Ral GDS RBD agarose (Millipore), anti-Rap1 (Millipore), anti-LFA-1 (M17/4; BD), anti-rat-FITC (BD), goat anti-mouse IgM/IgG1/IgG2a/IgE-AP (all Southern Biotech), Phalloidin-FITC (Sigma), recombinant mouse ICAM-1/Fc (R&D), CFSE (Molecular Probes), PP1 (Sigma), U73122 (Sigma), Wortmannin (Invivogen).

### Mice, cell culture and shRNA mediated RNAi

HPK1^−/−^ mice which carry a neomycin cassette in exon 1 disrupting protein expression were generated in the laboratory of Friedemann Kiefer (Max-Planck Institute for Molecular Biomedicine, Münster), backcrossed to the Balb/C genetic background >10 generations and were maintained in the animal facility at the University of Salzburg according to institutional and national guidelines for animal care and use; Wehi 231 and CH27 cells were maintained in RPMI 1640, 7.5% FCS (Gibco), 2 mM L-Glutamine, 50 U/ml penicillin, 50 mg/ml streptomycin and 50 µM 2-ME (all PAA); HPK1 shRNA pLKO.1-puro vectors (Sigma) were tested by Fugene HD transfection (Roche) for best performance (Wehi 231: 5′-gctgaaactgttcgcttatat-3′; CH27: 5′-ccaatgaacaaattcttgctt-3′; control target SHC002: 5′-caacaagatgaagagcaccaa-3′) and stable clones were grown at 0.3 µg/ml (Wehi 231) or 0.4 µg/ml (CH27) puromycin (Sigma).

### BCR induced actin polymerization

Assays were performed as described [44]; briefly, cells (1×10^6^/sample) were washed, stimulated for the indicated time points with anti-IgM F(ab')2, immediately fixed (3.7% PFA), permeabilized (0.05% Saponin) and stained with Phalloidin-FITC (0.1 µM); mean Phalloidin-FITC fluorescence intensities were measured by flow cytometry and are presented as fold F-actin increases compared to samples at t = 0.

### Static B-cell adhesion, spreading and inhibitor treatment

Static adhesion assays were performed as described [13]; fluorescence of adhering cells is displayed as % remaining fluorescence after 5 consecutive washing steps [Fl_rem._/Fl_tot_.×100]; for spreading assays, cells were preincubated with PP1 (100 µM), Wortmannin (100 nM), U73122 (1 µM) or DMSO for 30 min on 37°C and seeded onto ICAM-1/Fc layers for additional 30 min; phase contrast images were taken on a Nikon Eclipse microscope to determine the percentage of spread (phase dark) cells by counting four random fields.

### Immunoprecipitation and Rap1-GTP pulldown

Cells (5×10^6^/sample) were lysed in 1% Triton X-100, 10 mM Tris (pH 7.5), 150 mM NaCl, 5 mM EDTA, 10 mM NaF lysis buffer supplemented with protease (complete Mini; Roche) and phosphatase (Phosstop; Roche) inhibitors; lysates were precleared by centrifugation and subjected to immunoprecipitation for 3 h at 4°C; precipitates were washed, boiled in reducing loading buffer and separated on 4–12% gradient SDS-PAGE gels (BIORAD); Rap1-GTP pulldowns were performed with Ral GDS RBD agarose according to manufacturers instructions (Millipore); for quantification of Rap1-GTP amounts, co and kd band intensities were normalized for total Rap1 levels of lysates by densitometric evaluation (ImageJ) and all time points of Rap1-GTP samples were compared to the control target at t_0_ = 1.

### BCR internalization assay

BCR internalization was measured as previously described [45]. In brief, cells (1×10^6^/sample) were stained on ice with anti-IgM-FITC (5 µg/ml), shifted on 37°C for various time points and reactions were stopped by adding an excess of ice cold PBS/3% FCS; Mean fluorescence intensities (MFIs) of IgM^+^ cells were immediately analysed by flow cytometry and are displayed as % internalized receptor [(−MFI_t_/MFI_t0_×100)+100].

### Naïve B-cell proliferation assay

Splenic naïve B-cells were isolated untouched by depleting CD43^+^/CD4^+^/Ter119^+^ cells (B-cell isolation kit, Miltenyi); cells routinely reaching a purity of ≥98% were stained with CFSE (5 µM), seeded in triplicates in 96 well plates at a density of 2×10^5^ cells/well and were stimulated (25 µg/ml LPS, 5 ng/ml IL-4, 10 µg/ml anti-CD40, 5 µg/ml anti-IgM F(ab')2) or left untreated to determine proliferation after 72 h.

### Immunization

8 weeks old WT and HPK1^−/−^ mice (7 n each) were immunized intraperitoneally with 20 µg ovalbumin in alum/injection on day 0, 14 and 42 and ovalbumin-specific IgM, IgG1, IgG2a and IgE serum titers were analyzed (d21 and d49 depicted); control groups received PBS/alum alone, were analysed for equal ovalbumin unresponsiveness and used as unspecific serum controls for background subtraction in standard ELISA measurements.

## Supporting Information

Figure S1 Altered BCR dynamics in HPK1^−/−^ mice. (A) Anti-IgM-FITC-induced BCR internalization of WT and HPK1^−/−^ splenic IgM^+^ cells and (B) CH27 co and kd cells after various time points on 37°C; MFIs of FITC+ cells were analysed by flow cytometry and are displayed as % internalized receptor after 5 to 20 min on 37°C [(MFIt/MFIt0×100)+100]; graphs (A, B) show means ± SD; Student's t; n = 4. (C) HPK1 protein expression of CH27 wt, co and kd cells determined by Western blotting; ns, not significant.
(0.10 MB TIF)

Figure S2 Balb/C HPK1^−/−^ mice show normal T-cell dependent antibody responses. Mice (7 n/group) were injected intraperitoneally with 20 µg ovalbumin emulsified in alum on day 0, 14 and 42 and were analyzed for specific serum titers (d21/d49 depicted). No statistically significant difference (Student's t) for IgM, IgG1, IgG2a and IgE could be detected by ELISA measurement.
(0.13 MB TIF)

Figure S3 CFSE proliferation controls. Naïve splenic B-cells were CFSE labelled and stimulated with LPS (25 µg/ml), anti-CD40 (10 µg/ml) or anti-IgM F(ab')2 (5 µg/ml) for 72 h to measure control proliferation.
(0.10 MB TIF)
