# Supplementary figures and images for: HPK1 Associates with SKAP-HOM to Negatively Regulate Rap1-Mediated B-Lymphocyte Adhesion
Source: PLoS One. 2010 Sep 1;5(9):e12468. doi: 10.1371/journal.pone.0012468 (PMC2931690; doi:10.1371/journal.pone.0012468)

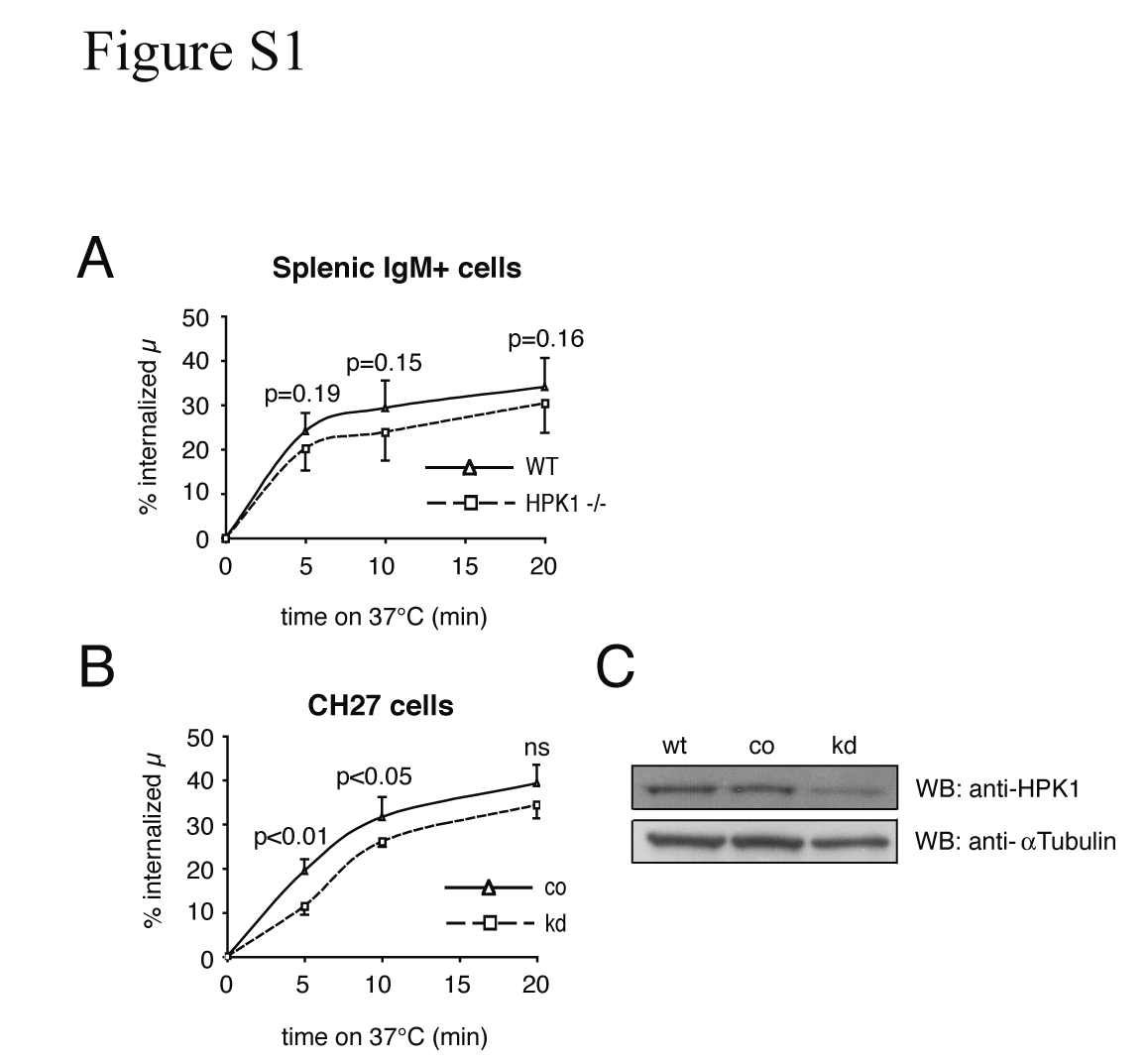

Supplement: Figure S1 — Altered BCR dynamics in HPK1−/− mice. (A) Anti-IgM-FITC-induced BCR internalization of WT and HPK1−/− splenic IgM+ cells and (B) CH27 co and kd cells after various time points on 37°C; MFIs of FITC+ cells were analysed by flow cytometry and are displayed as % internalized receptor after 5 to 20 min on 37°C [(MFIt/MFIt0×100)+100]; graphs (A, B) show means ± SD; Student's t; n = 4. (C) HPK1 protein expression of CH27 wt, co and kd cells determined by Western blotting; ns, not significant. (0.10 MB TIF) [file pone.0012468.s001.tif]

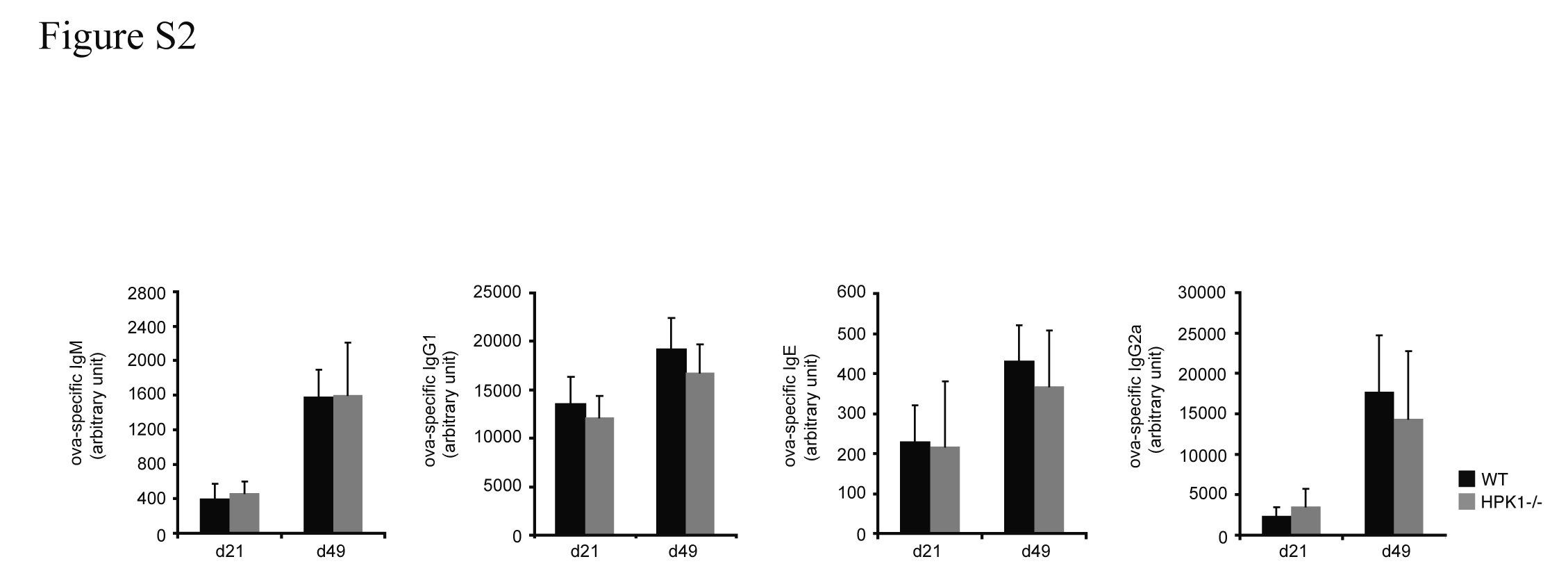

Supplement: Figure S2 — Balb/C HPK1−/− mice show normal T-cell dependent antibody responses. Mice (7 n/group) were injected intraperitoneally with 20 µg ovalbumin emulsified in alum on day 0, 14 and 42 and were analyzed for specific serum titers (d21/d49 depicted). No statistically significant difference (Student's t) for IgM, IgG1, IgG2a and IgE could be detected by ELISA measurement. (0.13 MB TIF) [file pone.0012468.s002.tif]

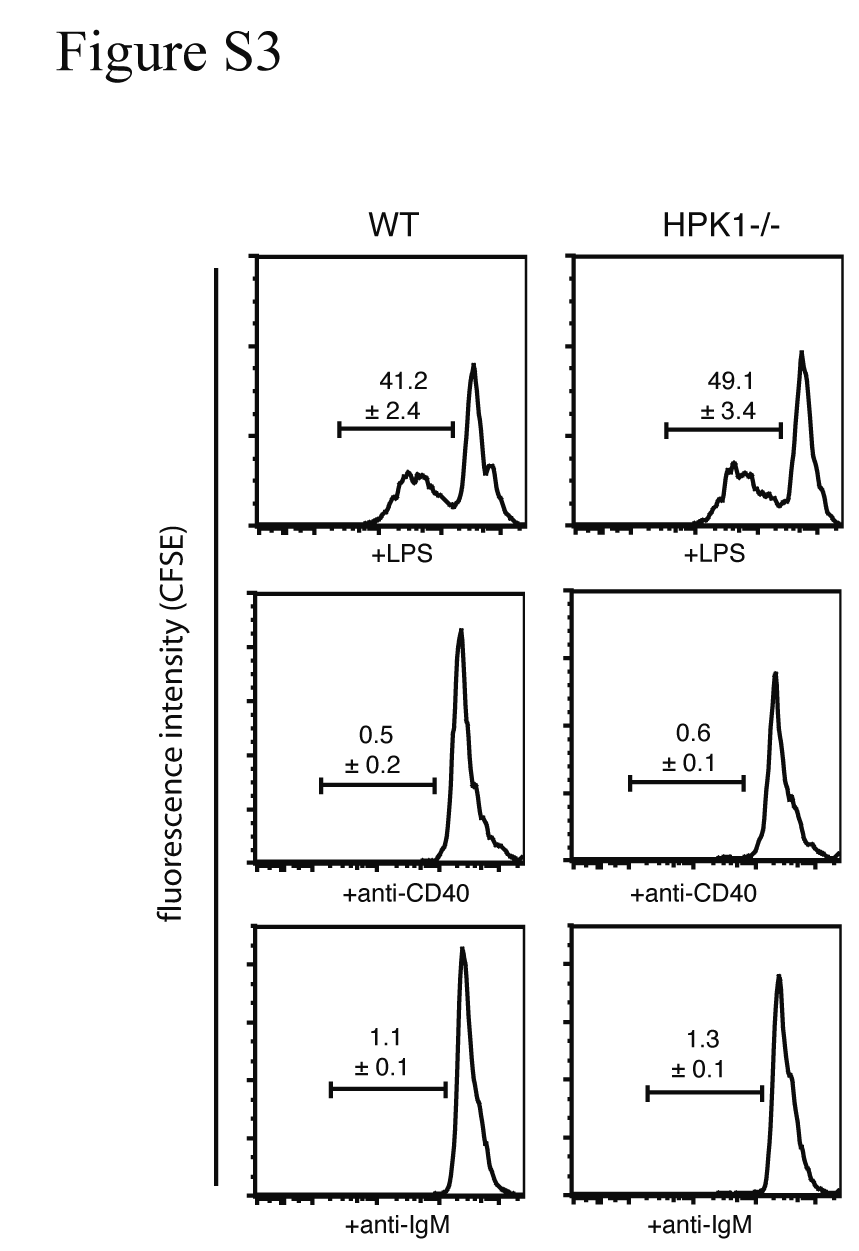

Supplement: Figure S3 — CFSE proliferation controls. Naïve splenic B-cells were CFSE labelled and stimulated with LPS (25 µg/ml), anti-CD40 (10 µg/ml) or anti-IgM F(ab')2 (5 µg/ml) for 72 h to measure control proliferation. (0.10 MB TIF) [file pone.0012468.s003.tif]
